# Sexually-Relevant Visual and Chemosensory Signals Induce Distinct Behaviors and Neural Activation Patterns in the Social African Cichlid, *Astatotilapia burtoni*

**DOI:** 10.3389/fnbeh.2018.00267

**Published:** 2018-11-21

**Authors:** Karen E. Field, Christopher T. McVicker, Karen P. Maruska

**Affiliations:** Department of Biological Sciences, Louisiana State University, Baton Rouge, LA, United States

**Keywords:** behavior, brain, multimodal, olfaction, sensory, social decision-making network, teleost, vision

## Abstract

Across vertebrates, the use of multimodal (multiple sensory modalities) signals has evolved to convey important information to receivers. Information content of multimodal signals can be the same as or different from information in each unimodal signal, and are classified as redundant or non-redundant, respectively, based on receivers’ behavioral responses. Despite the prevalence and importance of multimodal signaling across taxa, relatively little is known about how and where these signals are processed in the brains of receivers. We used the social African cichlid fish, *Astatotilapia burtoni*, to investigate how sexually-relevant visual and chemosensory uni- and multimodal signals from gravid (full of eggs) females influence behavior, brain activation patterns, and physiology in dominant males. We presented both visual and chemosensory signals either alone or together and found that males need sexually-relevant visual signals to engage in stereotypical courtship behaviors such as body quivers, waggles, and leads into spawning territories. However, the number of courtship behaviors was greater when males were exposed to multimodal visual-chemosensory signals, compared to either unimodal signal alone. When a female visual signal was absent, males increased swimming and overall activity in response to female-conditioned water compared to control water, suggesting that female-released chemosensory signals may stimulate male searching behavior and motivation. Importantly, we also tested anosmic (olfactory ablated) males to demonstrate that this behavior is primarily mediated by the olfactory system rather than gustation. Using the immediate early gene *cfos* as a proxy for neural activation, we also demonstrate differential activation in social and olfactory-relevant brain regions of dominant males exposed to unimodal and multimodal visual-chemosensory signals. We found at least one region that is preferentially activated by reception of signals from each sense, as well as regions that exhibit an additive effect on activation with multimodal visual-chemosensory stimulation. These data provide insight on how multimodal signals are processed in the brain and integrated with internal physiology of receivers to produce social behaviors, and lay the groundwork for future studies on the evolution of sensory perception.

## Introduction

Across taxa, animals must constantly assess their environment to make behavioral decisions. Signals sent via different sensory modalities, such as visual, chemosensory, mechanosensory, touch, and sound are often delivered together and reception of this information by a receiver is integrated with the animal’s own internal physiology to elicit context-dependent behaviors (Bradbury and Vehrencamp, 2000). This use of multimodal communication is prevalent across vertebrates, particularly during reproduction, providing receivers with varying types of information about the signaler such as breeding condition, motivation, and fitness qualities. Despite the importance of multimodal communication for survival and reproductive success, our understanding of how different sensory signals are processed in the brain of receivers to produce specific behavioral outputs is limited (Partan and Marler, 2005; Ronald et al., 2012).

The use of visual-chemosensory multimodal communication is widespread, with numerous examples from both invertebrate and vertebrate taxa (Kotrschal, 2000; Isogai et al., 2011; Mori, 2014). While vision is often the dominant sense mediating reproductive behaviors, chemosensory communication is also commonly used across the animal kingdom. It is particularly prevalent in fishes, where it functions in prey detection, predator avoidance, and social communication (Hara, 1994; Kotrschal, 2000). For example, females are often senders of potent chemical signals that provide important information for coordinating reproductive events, and in several fish species, these chemosensory signals can induce robust reproductive behavioral responses in male receivers (Stacey, 2011). However, the neural links between multisensory inputs and receiver behavioral output remains poorly understood (Ronald et al., 2012; Partan, 2013). Further, the physiology and/or reproductive state of receivers can influence how such sensory signals are processed (Insel, 2010). Thus, examining communication from a perspective that goes beyond behavioral responses to include receiver physiology and neural processing mechanisms is crucial for understanding the function and evolution of context-dependent signaling.

We used the social African cichlid fish, *Astatotilapia burtoni*, to investigate how visual and chemosensory signals alone and in combination from reproductively-receptive females influence the behavior, brain activation patterns, and hormonal responses of dominant males. *A. burtoni* is ideally suited for this inquiry because dominant males engage in elaborate, specific courtship behaviors in the presence of receptive females that includes sending information via multiple sensory channels (Maruska and Fernald, 2018), and males alter their courtship efforts based on distinguishing receptive from non-receptive females (Fernald and Hirata, 1977). Importantly, both males and females actively control urine release as a means of social communication in both aggressive and reproductive contexts, providing evidence for true chemosensory communication in this species (Maruska and Fernald, 2012; Field and Maruska, 2017). During reproduction, males increase urination in the presence of receptive females, while receptive females also increase urine release towards courting dominant males. Thus, while vision is the main sensory modality for communication in *A. burtoni*, chemosensory signals provide additional information for both males and females to modify context-dependent social decisions (Maruska and Fernald, 2012; Field and Maruska, 2017). How unimodal and multimodal signals from these two senses influence male behavior, physiology and brain activation patterns in socially-relevant nuclei, however, remains unexplored in this and the majority of fish species.

How relevant sensory and social information is integrated with an animal’s own internal physiology to elicit context-specific behaviors is a key goal of behavioral neuroscience (Insel, 2010). The social decision making network (SDMN) is a collection of highly conserved brain nuclei proposed as a framework for examining where and how this information leads to adaptive behaviors (Newman, 1999; O’Connell and Hofmann, 2011), but it is increasingly clear that many other brain regions outside of this network are also involved in social decisions. Our current knowledge of how multimodal sensory signals are processed in the brain of receivers to induce behavioral responses is limited. By associating specific behavioral output with neural activation patterns of receivers in response to unimodal and multimodal signals, we can help bridge this knowledge gap in social neuroscience. Further, little is known about where sexually-relevant chemosensory signals are processed in the brain above primary olfactory processing regions, especially in fishes (Nikonov and Caprio, 2005; Yaksi et al., 2009; Yabuki et al., 2016). Thus, by examining neural activation patterns in socially-relevant brain regions as a complement to behavioral responses in receivers, we can provide a framework to expand the current knowledge of the neural substrates that link sensory inputs to behavioral outputs.

To investigate how sexually-active males respond to unimodal and multimodal visual-chemosensory reproductive signals, we exposed dominant courting males to visual and chemosensory signals from gravid females either alone or combined, and recorded males’ behavioral and physiological responses. Further, we used *in situ* hybridization for the immediate early gene *cfos* to test for differences in neural activation of relevant brain nuclei in males receiving unimodal and multimodal signals from receptive females. This approach allows us to elucidate the brain regions important for processing visual and chemosensory signals alone, and those involved in integrating information from both sensory channels when presented together in a naturalistic reproductive context.

## Materials and Methods

### Experimental Animals

Adult *Astatotilapia burtoni* (Günther, 1894) from a wild-caught stock were kept in aquaria under water and lighting conditions that are similar to their natural habitat in Lake Tanganyika, Africa (28°C; pH 8.0; 12 h light:12 h dark cycle). These fish were bred in laboratories since original collection in the 1970s and exhibit behaviors similar to those in wild populations (Fernald and Hirata, 1977). Aquaria contained gravel-covered floors and halved terra cotta pots to serve as shelters and spawning territories. Fish were fed cichlid flakes (AquaDine, Healdsburg, CA, USA) daily and supplemented with brine shrimp twice a week. All experiments were performed in accordance with the recommendations and guidelines provided by the National Institutes of Health Guide for the Care and Use of Laboratory Animals, 2011. The protocol was approved by the Institutional Animal Care and Use Committee (IACUC) at Louisiana State University, Baton Rouge, LA, USA.

### Experimental Protocol

**Figure 1:**
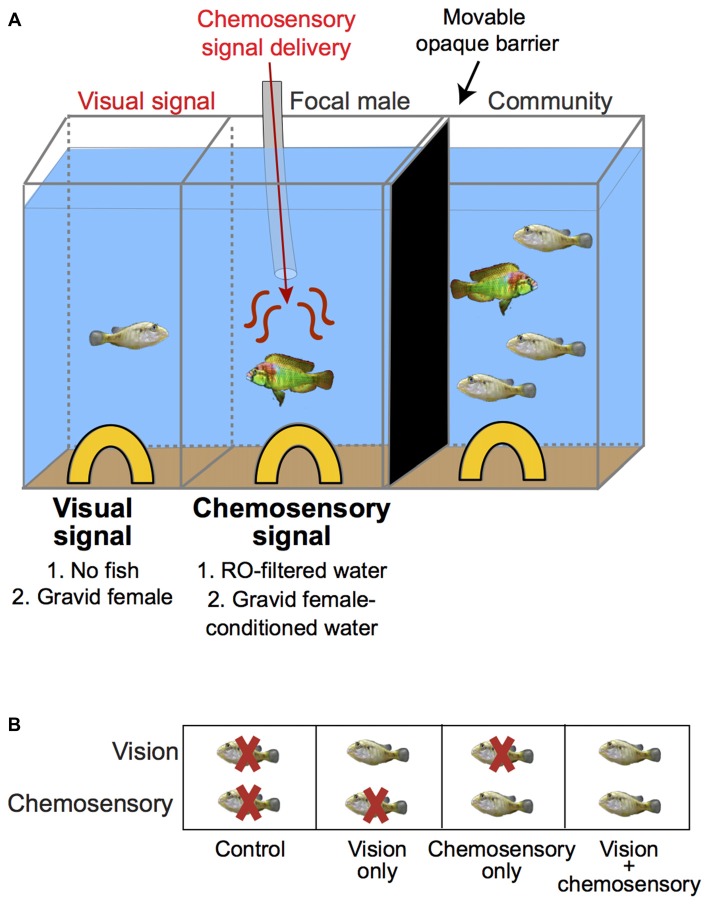
Experimental paradigm to examine behavioral responses and neural activation patterns in dominant male *A. burtoni* exposed to unimodal and multimodal visual and chemosensory signals from females. **(A)** Focal males were acclimated to the center compartment with chemosensory stimulus delivery tube present. A movable black barrier visually blocked the left visual stimulus compartment while allowing visual exposure to a fish community consisting of one male and three females in the right compartment for 24–48 h before experiments. Immediately before experiments, the black barrier was moved to visually block the community on the right and expose the left compartment. Visual signals were presented in the left compartment and chemosensory signals were simultaneously delivered to the center compartment via a gravity-feed bottle at a controlled flow rate. **(B)** The combinations of visual and chemosensory stimuli presented to males are shown with terminology used to describe each condition indicated below boxes: “Control” refers to no fish/reverse osmosis (RO)-filtered water, “Visual only” refers to gravid female/RO-filtered water; “Chemosensory only” refers to no fish/gravid female-conditioned water, and “Visual and chemosensory” refers to gravid female/gravid female-conditioned water.

Dominant males (standard length (SL): 43.19 ± 2.19 mm (mean ± SD)) were housed in aquaria in mixed broods prior to being selected for experiments. Experiments were conducted in 37.85 L aquaria that were divided into three equal sized compartments (16.7 × 25.3 × 30.8 cm each) by clear, acrylic barriers permanently sealed into the tank (Figure 1) and verified to block transmission of water and chemosensory cues as in our previous study (Field and Maruska, 2017). Each compartment contained a layer of gravel at the bottom, an air stone, and a territory/shelter (half terracotta pot). All experimental compartments were drained, cleaned, and refilled between experiments to ensure no cross-experiment contamination of odorants. Prior to experiments, focal dominant males were selected based on bright coloration and behaviors typical of dominance such as defending territories and actively courting females for at least three consecutive days. Males were then moved to the center experimental compartment and allowed to acclimate for 48 h while visually exposed to a community consisting of one male (smaller than focal dominant male) and three females in the right compartment while a movable black opaque barrier visually blocked the empty left compartment. The center experimental compartment contained the chemosensory delivery tube throughout the entire acclimation period of the focal male.

To examine responses of focal males to unimodal and multimodal visual and chemosensory signals, we presented visual and chemosensory stimuli in different combinations. Visual signals were provided in the left compartment and consisted of the presence of a gravid female or no fish (empty compartment). Chemosensory signals were delivered through a tube connected to a gravity feed bottle and consisted of 850 mL of gravid female-conditioned water or 850 mL of reverse osmosis (RO)-filtered water as a control (see below). The flow rate of the chemosensory stimulus was tested the day before and morning of experiments and was verified to be 0.325 ± 0.25 L/min. The stimuli were presented in the following combinations (visual in left compartment/chemosensory in center compartment): (1) no fish/RO-filtered water (termed “control”); (2) gravid female/RO-filtered water (termed “vision only”); (3) no fish/female-conditioned water (termed “chemosensory only”); and (4) gravid female/female-conditioned water (termed “vision and chemosensory”). For clarity, Figure 1B outlines terminology of the stimulus delivery used throughout this study.

To determine whether focal male responses were mediated by olfaction or gustation (taste), we also tested anosmic (ablation of olfactory sense) focal males in the same experimental paradigm in the chemosensory only condition. Males (*n* = 3; SL: 43.2 ± 0.70 mm; gonadosomatic index (GSI) > 0.70) were rendered anosmic 2 days prior to behavioral experiments by sedating and immobilizing the fish via gradual cooling in ice-cold cichlid-system water and then bilaterally severing the olfactory nerves between the olfactory epithelia and olfactory bulbs. Anosmia was verified by lack of *cfos* expression in the olfactory bulb and reduced *cfos* expression in the posterior nucleus of the dorsal telencephalon (Dp), a forebrain region important in olfactory processing. Three sham-handled males were included to ensure effects in anosmic males were not due to the handling procedure. Sham males had the tissue covering their olfactory nerve cut as anosmic males did, but without causing any damage to the olfactory nerve. Incisions for both anosmic and sham-handled fish were sealed with Vetbond™. For behavioral testing, anosmic and sham males received only chemosensory signals from gravid females (no visual signal). The behavior of sham-handled males was verified as not different from intact males exposed to the same sensory conditions (chemosensory stimulus only).

Gravid (ripe with eggs) females (SL: 37.5 ± 2.1 mm, GSI > 7.0) used as visual stimuli were selected the morning of experiments prior to feeding based on the presence of a distended abdomen due to the presence of large ova. GSI ([gonad mass/(body mass-stomach mass)*100]) and ovulation state of females were verified after experiments. For female-conditioned water used for chemosensory stimuli, four gravid females were selected in the morning prior to feeding and placed in a bucket with an air stone and allowed to soak in RO water for 5 h. All solid materials (feces and/or algae) were removed from the water before stimulus delivery. The control RO-filtered water stimulus was also soaked for 5 h in an identical (but separate) bucket with an air stone for 5 h to match handling of female-conditioned water.

On the morning of experiments (08:30–09:30 a.m.), the focal male and visual stimulus female (if present) were fed two flakes of cichlid food, and the chemosensory stimuli were prepared. Following the 5 h. soaking period (at 1:30–2:30 p.m.), the gravid females (if present) were removed from the soaking bucket, and the stimulus water was transferred into a gravity feed bottle. The black barrier was simultaneously removed and placed between the experimental and community compartments such that the focal male was visually exposed to the left stimulus compartment and visually blocked from the community on the right. Experiments lasted 40 min from the start of chemosensory stimulus delivery.

### Behavioral Quantification

Focal male behaviors during the first 15 min of each experiment were scored using BORIS software^1^. Only the first 15 min were quantified because behaviors declined after this point and were therefore not representative of the stimulus-evoked response of the focal male. The male behaviors quantified were number of courtship behaviors and time spent performing searching behavior (increased swimming activity/arousal). Courtship behaviors included body quivers, tail waggles, and leads towards the shelter. Searching was defined by increased swimming speed with at least one change in direction in the water column (up or down) that lasted at least 3 s. Bouts of searching were separated by pauses in swimming that lasted at least 2 s.

### Tissue Preparation

Focal males were collected after 40 min of stimulation, sedated and immobilized in ice-cold cichlid-system water, and sacrificed by rapid cervical transection. SL, body mass, stomach mass, gonad mass and GSI were recorded. All dominant focal males had a GSI > 0.70 and visual stimulus females had a GSI > 7.0. Blood was collected from the caudal vein and centrifuged at 8,000 rpm for 10 min to isolate serum, and then stored at −80°C. Brains were removed and fixed overnight in 4% paraformaldehyde (PFA) made in 1× PBS, rinsed overnight in 1× PBS, and cryoprotected in 30% sucrose for 1–5 nights prior to sectioning (all at 4°C). Brains were embedded in OCT media (TissueTek), sectioned coronally at 20 μm with a cryostat, collected on alternate charged slides (Superfrost Plus, VWR), dried overnight at room temperature, and stored at −80°C.

### *In situ* Hybridization for *cfos*

To examine differences in neural activation in the brains of behaving focal males, we performed colorimetric *in situ* hybridization for the immediate early gene *cfos* using riboprobes specific for *A. burtoni cfos* mRNA as previously described (Butler and Maruska, 2016). We chose *cfos* as a marker for this study because our focus was to examine which brain regions received unimodal and multimodal visual-chemosensory inputs, rather than the brain regions involved in the expression of behavioral outputs. It was previously shown in zebrafish that *cfos* is an ideal marker for determining neural activity associated with processing perceptual stimuli from the social environment (Teles et al., 2015). Briefly, slides of sectioned brains were rinsed at room temperature in 1× PBS, fixed with 4% PFA, rinsed with 1× PBS, treated with proteinase K (10 μg/mL), rinsed with 1× PBS, fixed with 4% PFA, rinsed with 1× PBS followed by milliQ water, treated with 0.25% acetic anhydride in 0.1 M triethanolamine-HCL (pH 8.0), and rinsed with 1× PBS. Slides were then incubated in warmed pre-hybridization buffer at 60–65°C for 3 h. Subsequently, slides were then incubated with warmed hybridization buffer containing *cfos* riboprobe at 60–65°C for 12–16 h sealed with HybriSlip covers in sealed humidified chambers. Then, at this same temperature, sections were washed in 2× SSC in 50% formamide with 0.1% Tween-20, followed by a 1:1 mixture of 2× SSC and maleic acid buffer with 0.1% Tween-20 (MABT). MABT washes were then performed again at room temperature. Non-specific binding was blocked with MABT with 2% bovine serum albumin (BSA) for 3 h and then slides were incubated with alkaline phosphatase (AP) anti-DIG fragments (Sigma Aldrich) overnight at 4°C in a sealed humidified chamber. Slides were then rinsed in MABT at room temperature, incubated in AP buffer, and then developed with nitro-blue tetrazolium/5-bromo-4-chloro-3′-indolyphosphate (NBT/BCIP; Sigma Aldrich) substrate at 37°C in darkness for 2–3 h. Slides were then rinsed in 1× PBS, fixed in 4% PFA, washed in 1× PBS, and coverslipped with aquamount aqueous mounting media (Fisher Scientific).

### Quantification of *cfos*-Expressing Cells

To quantify differences in *cfos* staining in the brain, slides were visualized on a Nikon Eclipse Ni microscope and photographed with a color digital camera controlled by Nikon NIS-Elements software. Brightfield and phase contrast were used to visualize neuroanatomical markers and brain nuclei in relation to stained cells. Quantifications were done by an observer blind to experimental condition. *cfos*-positive cells were easily identifiable by dark purple staining inside the cell with a clear, discernible border. Final images were adjusted for levels, contrast, and brightness in Adobe Illustrator CC v21.10. Neuroanatomical structures were identified using a cresyl violet stained *A. burtoni* reference brain and *A. burtoni* brain atlas. Stereotactic and neuroanatomical markers were used to designate the borders and rostro-caudal extent of each region to ensure consistency across animals. The following socially-relevant regions of the brain were quantified: ventral nucleus of the ventral telencephalon (Vv), supracomissural nucleus of the ventral telencephalon (Vs), dorsal part of the ventral telencephalon (Vd), granular zone of lateral part of the dorsal telencephalon (Dl-g), fourth and fifth subdivisions of central part of the dorsal telencephalon (Dc-4 and Dc-5), anterior tuberal nucleus (ATn), anterior part of the periventricular preoptic area (nPPa). The posterior nucleus of the dorsal telencephalon (Dp) was also quantified, as it is an important olfactory processing region. Images were taken at the highest magnification (10× or 20× objective) that encompassed the entire area of interest. For 10× images (Vs, Dl-g, Dp and ATn), nuclei borders were outlined with either 50 μm × 50 μm gridlines (Dp, ATn) or 80 μm × 80 μm gridlines (Vs, Dl-g) applied to each image. *cfos*-expressing cells in five randomly chosen boxes per section were counted for Vs and Dl-g and four randomly chosen boxes for Dp and ATn and cell density calculated by dividing the number of cells within the boxes by the total area of the boxes. For 20× images (Vv, Vd, nPPa, Dc-4 and Dc-5), the same procedure was followed, except nuclei borders were overlaid with 50 μm × 50 μm grid lines and *cfos*-expressing cells were counted in three boxes. For all regions, four consecutive sections were quantified for each region and averaged together for a cell density value (#cells/μm^2^) of that region for each animal. Alternate sections were used for quantification so that adjacent 20 μm sections were separated by 40 μm (and cell diameters are ~<10–25 μm on average) ensuring no double counting of cells. Density values were then averaged across individuals exposed to the same sensory stimulus conditions.

### Steroid Hormone Assays

To test for differences in circulating sex-steroid hormones among visual-chemosensory conditions, plasma 11-ketotestosterone (11-KT) and estradiol (E_2_) were measured using Enzyme ImmunoAssay (EIA) kits (Cayman Chemical Inc.), as previously described and validated for *A. burtoni* (Maruska and Fernald, 2010b). For both steroids, 4.4 μl of plasma from each focal male was extracted three times using 220 μl of ethyl ether and evaporated under a fume hood prior to re-constitution in assay buffer (1:50 dilution). Kit protocols were then strictly followed, plates were read at 405 nm using a spectrophotometer microplate reader and steroid concentrations determined based on standard curves.

### Statistical Analysis

Behavior and neural activation data were compared with one-way ANOVAs. Focal male courtship and searching behavior could not be normalized by transformation and were compared with non-parametric Kruskal-Wallis (KW) one-way ANOVA on Ranks with Dunn’s *post* *hoc* tests (α =0.05). Behavior and neural activation in anosmic and intact males were compared using student’s *t*-tests. To compare searching behavior in anosmic and intact males, data were log-transformed to pass equal variance. To test for differences in neural activation across sensory stimulus conditions, the density of *cfos* expressing cells in each brain nucleus was compared with parametric one-way ANOVA with SNK *post hoc* tests. Cell density data that could not be transformed (one brain region only, Dc-5) were compared with KW one-way ANOVA on Ranks with Dunn’s *post hoc* test. Pearson correlation tests were used to test for relationships among *cfos*-labeled cell densities across brain regions, and with courtship and search behavior to generate co-activation heat maps for each stimulus combination. Factor analyses were done using principal component extractions with Eigenvalues >1 and components plotted in rotated space (varimax rotation). For discriminant function analysis (DFA), any missing values were replaced with the group mean (Dc-5 only). Significant outliers detected by Grubb’s test were removed prior to all comparisons (Dc-5: one outlier removed from *cfos* quantification). Steroid hormone levels were analyzed across stimulus condition for intact and anosmic males using ANCOVA with body size as a covariate. Statistical comparisons were performed in SigmaPlot 12.3 or SPSS 24.

## Results

### Behavioral Response to Unimodal and Multimodal Visual and Chemosensory Signals

**Figure 2:**
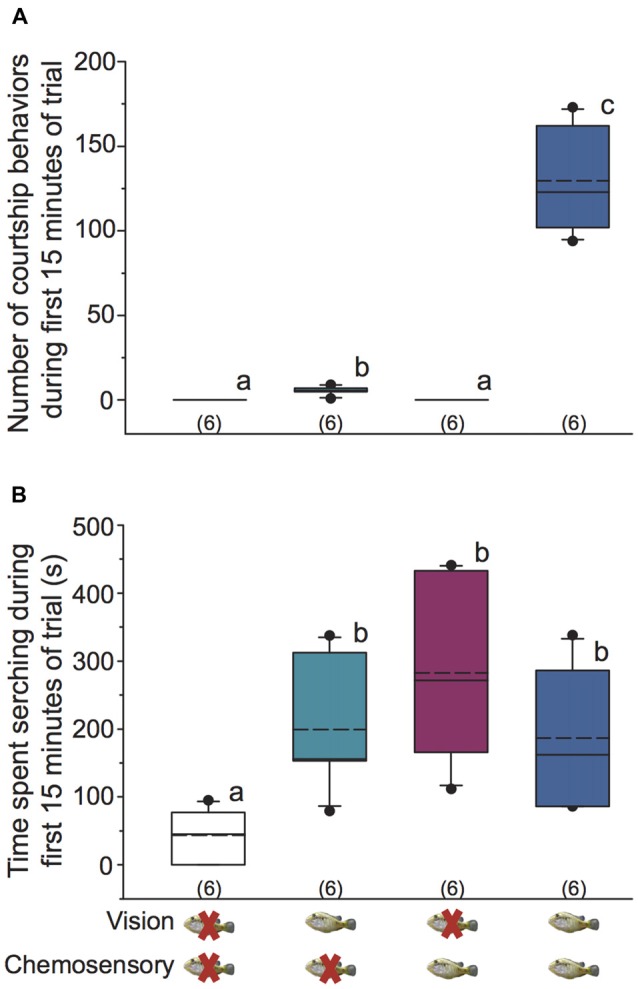
Visual and chemosensory signals from gravid females induce distinct behaviors in dominant *A. burtoni* males. **(A)** Focal males require a visual signal from females to engage in courtship behaviors, but visual signals alone are sufficient to induce some courtship. Male courtship behaviors are dramatically increased when both visual and chemosensory signals from females are present. **(B)** Focal males spend more time searching when a visual, chemosensory, or paired visual-chemosensory signal from females is present compared to control conditions. Different letters indicate statistical differences at *P* < 0.05 (Kruskal-Wallis (KW) one-way ANOVA on Ranks, SNK *post hoc*). Box plots were used to represent data: solid line indicates the median and dashed line indicates the mean. The box extends to the furthest data points within the 25th and 75th percentile, and whiskers extend to the 10th and 90th percentile. Closed circles indicate data points outside the 5th and 95th percentile. Sample sizes are shown in parentheses.

Focal males did not perform any courtship behaviors (body quivers, tail waggles, leads to shelter) in control and chemosensory only conditions (Figure 2A). In vision only conditions, focal males performed more courtship behaviors (0–9 total courtship behaviors) than both control and chemosensory only conditions. However, the number of courtship behaviors was significantly higher when focal males received visual and chemosensory signals together (KW one-way ANOVA on Ranks, *H* = 22.212, *df* = 3, *P* < 0.001; Dunn’s *P* < 0.05; Figure 2A). Thus, dominant *A. burtoni* males must see a receptive female to engage in specific courtship behaviors, but courtship is enhanced when visual signals are paired with chemosensory signals from females.

Searching behavior was observed in all focal males across all stimulus conditions. Focal males spent significantly more time searching in vision only, chemosensory only, and visual-chemosensory conditions compared to controls, but searching did not differ among the three non-control conditions (KW one-way ANOVA on Ranks, *H* = 12.645, *df* = 3, *P* = 0.005; Dunn’s *P* < 0.05; Figure 2B).

### Neural Activation

**Figure 3:**
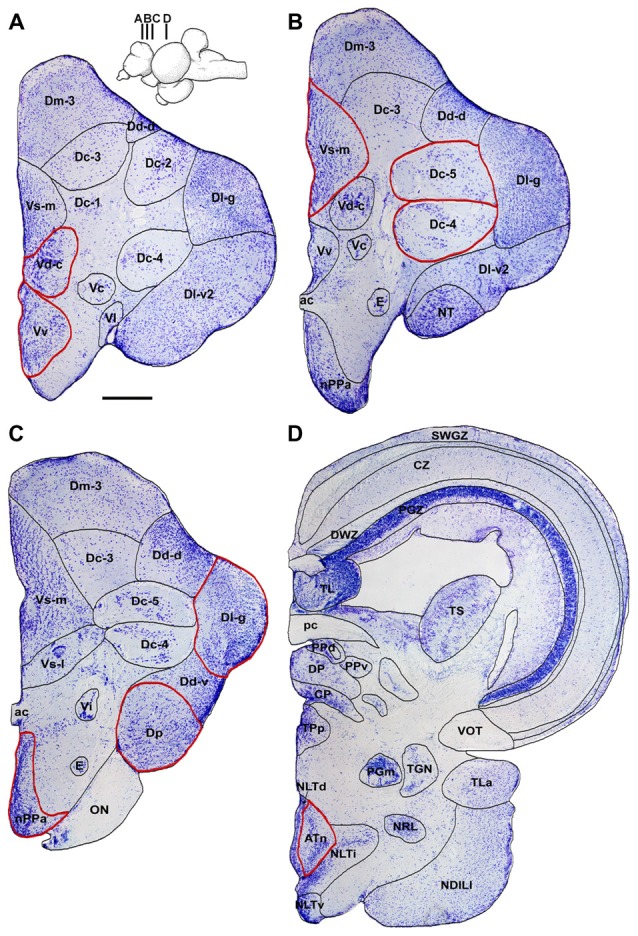
Representative transverse sections from the *A. burtoni* brain showing the locations of relevant nuclei quantified in this study. Cresyl violet-stained sections are shown from rostral **(A)** to caudal **(D)** and nuclei are outlined and labeled. Only the right half of the brain is shown for each, relevant quantified regions are outlined in red, and inset shows the approximate locations of each section on a sagittal brain. Scale bar = 250 μm. Abbreviations: ac, anterior commissure; ATn, anterior tuberal nucleus; CP, central posterior thalamic nucleus; CZ, central zone of tectum; Dc-1–5, central part of the dorsal telencephalon, subdivisions 1–5; Dd-d, dorsal part of the dorsal telencephalon, dorsal subdivision; Dd-v, dorsal part of the dorsal telencephalon, ventral subdivision; Dl-g, granular zone of lateral zone of the dorsal telencephalon; Dl-v2, ventral part of the lateral zone of the dorsal telencephalon, subdivision 2; Dm-3, medial part of the dorsal telencephalon, subdivision 3; Dp, posterior part of the dorsal telencephalon; DP, dorsal posterior thalamic nucleus; E, entopeduncular nucleus; NDILl, lateral part of the diffuse nucleus of the inferior lobe; NLTd, lateral tuberal nucleus, dorsal part; NLTi, lateral tuberal nucleus, intermediate part; NLTv, lateral tuberal nucleus, ventral part; nPPa, parvocellular preoptic nucleus, anterior part; NRL, nucleus of the lateral recess; NT, nucleus taenia; ON, optic nerve; pc, posterior commissure; PGm, medial preglomerular nucleus; PGZ, periventricular gray zone of tectum; PPd, dorsal periventricular pretectal nucleus; PPv, ventral periventricular pretectal nucleus; SWGZ, superficial gray and white zone of tectum; TGN, tertiary gustatory nucleus; TL, torus longitudinalis; TLa, nucleus of the torus lateralis; TPp, periventricular nucleus of the posterior tuberculum; TS, torus semicircularis; Vc, central part of the ventral telencephalon; Vd-c, dorsal part of the ventral telencephalon, caudal subdivision; Vi, intermediate nucleus of the ventral telencephalon; Vl, lateral part of the ventral telencephalon; VOT, ventral optic tract; Vs-l, lateral part of the supracommissural nucleus of the ventral telencephalon; Vs-m, medial part of the supracommissural nucleus of the ventral telencephalon; Vv, ventral part of the ventral telencephalon.

**Figure 4:**
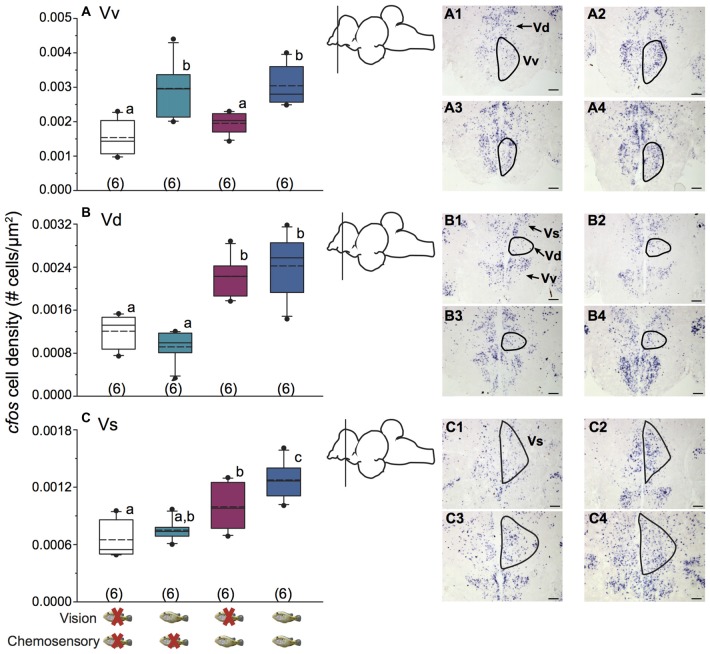
Unimodal and multimodal visual and chemosensory signals from females elicit distinct neural activation patterns in ventral telencephalic brain regions of dominant *A. burtoni* males. **(A)** Focal males show greater *cfos* expression in Vv when a visual signal is present, regardless of whether a chemosensory signal is present or not. **(B)** There is greater *cfos* expression in Vd when a chemosensory signal is present, regardless of whether a visual signal is present or not. **(C)** Neural activation in Vs is greater in the unimodal chemosensory signal condition compared to control, but it did not differ from the visual only condition. Activation in Vs was also greater in visual-chemosensory compared to all other conditions. Photos show representative examples of *cfos* staining in each region for control conditions (1), visual only (2), chemosensory only (3), and visual and chemosensory (4). Outlines demonstrate approximate quantified area for each region. Scale bars represent 100 μm. Schematics show a lateral view of the brain with the approximate location of each region. Different letters indicate statistical significance at *P* < 0.05 (one-way ANOVA). See Figure 2 for box plot descriptions.

**Figure 5:**
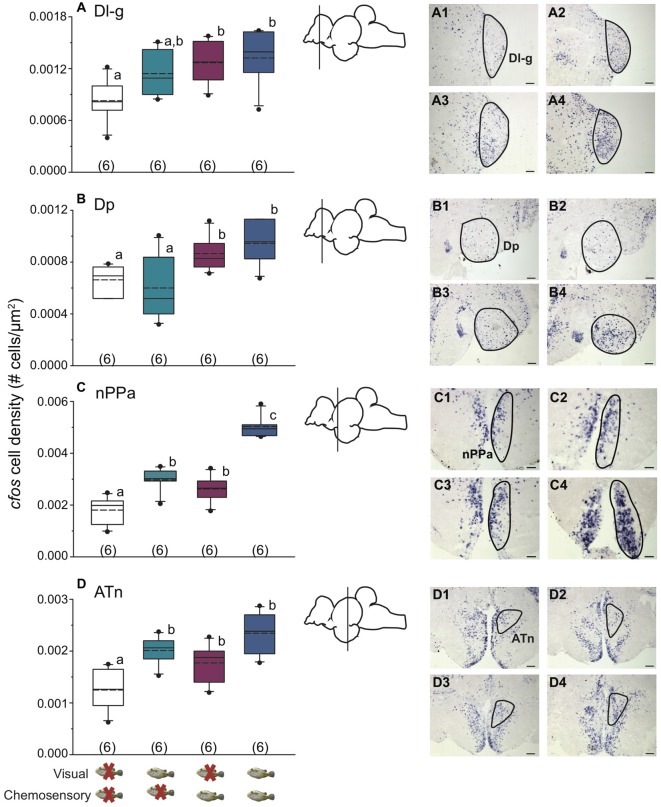
Unimodal and multimodal visual and chemosensory signals from females elicit distinct neural activation patterns in dorsal telencephalic and hypothalamic brain regions of dominant *A. burtoni* males. **(A)** In Dl-g, there is greater *cfos* expression in chemosensory only and visual-chemosensory conditions compared to controls, with activation in vision only conditions not different from either of these conditions or controls. **(B)** There is higher *cfos* expression in Dp when a chemosensory signal is present, regardless of whether a visual signal is present or not. **(C)** nPPa shows the greatest neural activation when both visual and chemosensory signals from females are present. **(D)** In ATn, focal males show higher *cfos* expression in all visual-chemosensory sensory conditions compared to controls. Photos show representative examples of *cfos* staining in each region for control conditions (1), visual only (2), chemosensory only (3), and visual and chemosensory (4). Outlines demonstrate quantified area for each region. Scale bar in **(A)** represents 50 μm. Scale bars in **(B–D)** represent 100 μm. Schematics show a lateral view of the brain with approximate location of each region. Different letters indicate statistical significance at *P* < 0.05 (one-way ANOVA). See Figure 2 for box plot descriptions.

Figure 3 shows representative low magnification cresyl violet-stained transverse sections from *A. burtoni* with locations of relevant regions quantified for neural activation (measured as *cfos* cell density) in this study. Focal males exposed to unimodal and multimodal visual and chemosensory signals from females showed differential neural activation patterns in regions that process sensory inputs and mediate social decisions. For example, activation in Vv was significantly higher when a visual signal was present regardless of whether or not a chemosensory signal was present (one-way ANOVA *F*_(3,20)_ = 8.516, *P* < 0.001; SNK *P* < 0.05; Figure 4A). Activation in Vd also showed differences depending on stimuli, with greater neural activation when a chemosensory signal was present whether or not a visual signal was present (one-way ANOVA *F*_(3,20)_ = 16.627, *P* < 0.001; SNK *P* < 0.05; Figure 4B). Activation in Vs was higher in the chemosensory only condition compared to the control, but did not differ from the visual only condition. Further, Vs activation was greater when multimodal visual-chemosensory signals were presented compared to all other conditions (one-way ANOVA *F*_(3,20)_ = 11.262, *P* < 0.001; SNK *P* < 0.05; Figure 4C). Activation in Dl-g in chemosensory only and visual-chemosensory conditions was greater than that in controls, but the visual only condition did not differ from either of these or the control condition (one-way ANOVA *F*_(3,20)_ = 3.317, *P* = 0.041; SNK *P* < 0.05; Figure 5A). Dp had greater activation when a chemosensory stimulus was present, regardless of whether or not a visual signal was present, compared to controls (one-way ANOVA *F*_(3,20)_ = 4.565, *P* = 0.014; SNK *P* < 0.05; Figure 5B). For nPPa, activation was greater when visual or chemosensory unimodal signals were present compared to the control. Further, when visual and chemosensory signals were presented together there was greater activation in nPPa compared to all other conditions (one-way ANOVA *F*_(3,20)_ = 41.205, *P* < 0.001; SNK *P* < 0.05; Figure 5C). In ATn, activation was greater in all stimulus conditions compared to the control, but stimulus conditions did not differ from one another (one-way ANOVA *F*_(3,20)_ = 7.967, *P* = 0.001; SNK *P* < 0.05; Figure 5D). There was a significant difference in activation in Dc-5 across sensory stimulus conditions, but *post hoc* tests were unable to detect differences (KW one-way ANOVA on Ranks, *H* = 9.778, *df* = 3, *P* = 0.021, Dunn’s *P* > 0.05). No significant differences in activation occurred among stimulus conditions in Dc-4 (one-way ANOVA *F*_(3,20)_ = 0.424, *P* = 0.738).

### Behavior and Neural Activation in Anosmic Males

**Figure 6:**
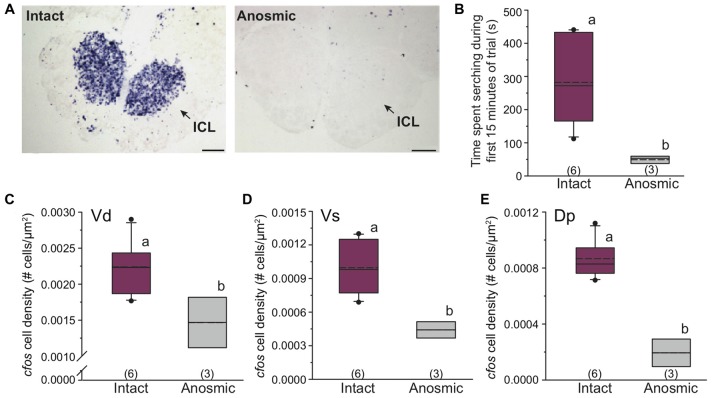
Anosmic dominant males show altered searching behavior and reduced *cfos* expression in known olfactory processing regions of the brain. **(A)** Anosmic males show decreased* cfos* expression (purple staining) in the olfactory bulb compared to intact males. Arrows indicate inner cellular layer of the olfactory bulb. **(B)** Anosmic males receiving only chemosensory signals from gravid females spend less time searching compared to intact males. Anosmic males also have reduced neural activation (*cfos* cell density) in Vd **(C)**, Vs **(D)** and Dp **(E)** compared to intact males receiving the same chemosensory only stimulus. Scale bars in **(A)** represent 100 μm. Different letters indicate statistical significance at *P* < 0.05. See Figure 2 for box plot descriptions.

We used anosmic males to test whether behavioral responses were mediated by olfaction or taste. Anosmia was verified in males by absence of *cfos* staining (no neural activation) in the inner cellular layer of the olfactory bulb, indicating no transmission of sensory information from the olfactory epithelium to the olfactory bulb when the olfactory nerves were severed (see Figure 6 for example *cfos* staining in the olfactory bulb). Anosmic males presented with only chemosensory signals from females (no visual signal) showed reduced *cfos* staining in the ICL compared to intact males that received the same stimulus (Figure 6A). Further, anosmic males spent less time searching compared to intact focal males (student’s *t*-test, *P* = 0.002; Figure 6B) and also had fewer *cfos-*stained cells in Vd (student’s *t*-test, *P* = 0.039) and Vs (student’s *t*-test, *P* = 0.009) as well as in Dp (a known olfactory processing region) compared to intact focal males (student’s *t*-test, *P* = 0.003; Figures 6C–E).

### Hormone Responses of Intact and Anosmic Males

We measured circulating levels of 11-KT and E_2_ in intact males exposed to uni- and multimodal visual and chemosensory signals, and in anosmic males. There was no difference in either hormone among males in any experimental group (11-KT: ANCOVA *F*_(5,22)_ = 1.203, *P* = 0.34; E_2_: ANCOVA *F*_(6,19) =_ 1.892, *P* = 0.135).

### Correlations and Multivariate Analyses of Brain Regions and Social Behaviors

**Figure 7:**
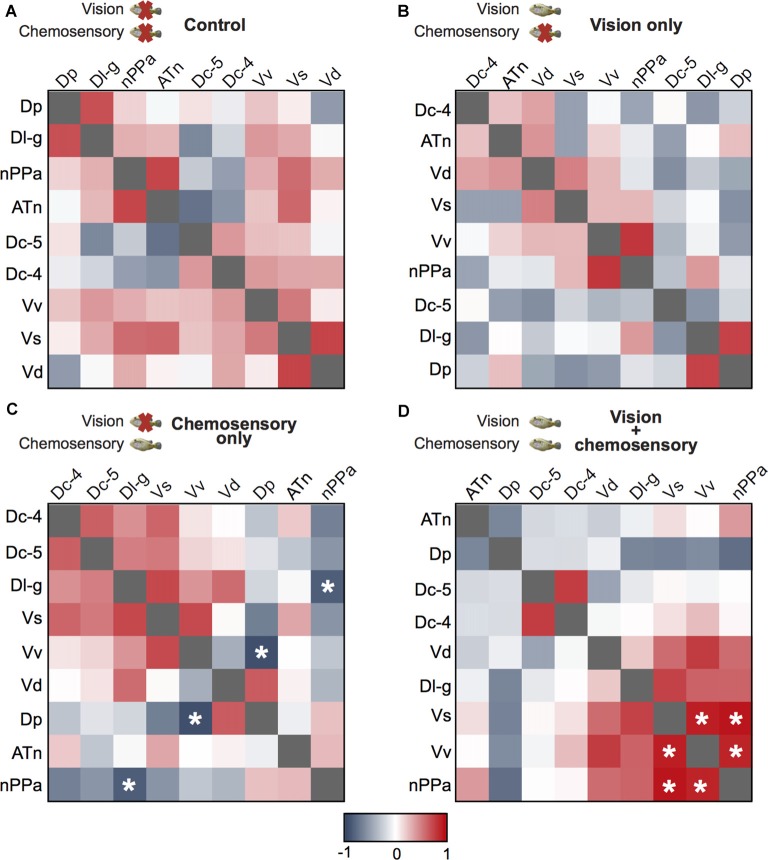
Males exposed to unimodal and multimodal visual and chemosensory signals from gravid females show different co-activation patterns in the brain. Heat maps of Pearson correlation coefficients (*R* = color scale) of *cfos* staining in brain nuclei in **(A)** control conditions; **(B)** unimodal visual only; **(C)** unimodal chemosensory only; and **(D)** multimodal visual and chemosensory. Heat maps ordered based on hierarchical clustering of brain regions for each condition. See Tables 1–4 for *R* and *P* values. Asterisks indicate significance at *P* < 0.05.

**Figure 8:**
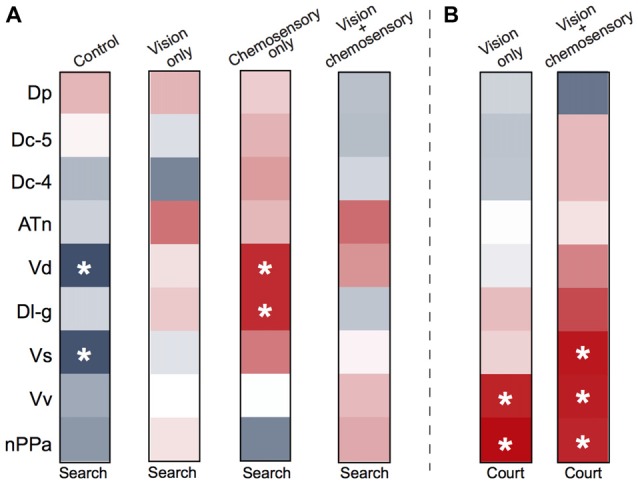
Males exposed to unimodal and multimodal visual and chemosensory signals from gravid females show different neural co-activation patterns related to social behaviors. Heat maps of Pearson correlation coefficients (*R* = color scale of Figure 6) of *cfos* staining in brain nuclei and **(A)** searching behaviors across all sensory conditions, and **(B)** courtship behaviors in unimodal visual only and multimodal visual and chemosensory conditions. Control and unimodal chemosensory conditions are not included in **(B)** because no courtship behaviors occurred in these conditions. See Tables 5, 6 for *R* and *P* values. Asterisks indicate significance at *P* < 0.05.

To investigate functional connectivity of the examined brain regions and how it relates to expression of social behaviors, we created heat maps from Pearson correlation coefficients of *cfos* cell density (Figure 7) and number of social behaviors for each sensory condition (Figure 8). In control and visual only conditions, there were no significant correlations between any brain regions (Figures 7A,B). In the visual only condition, courtship behavior positively correlated with activation in nPPa and Vv (Figure 8). When only chemosensory signals were present, negative correlations were observed between activation in Vv and Dp as well as between nPPa and Dl-g (Figure 7C). However, there was a positive correlation between searching behavior and neural activation in both nPPa and Dl-g (Figure 8A). When both visual and chemosensory signals were present, Vv, Vs and nPPa were positively correlated with each other (Figure 7D) and with courtship behaviors (Figure 8B). Tables 1–4 show Pearson correlation coefficients and *P* values for *cfos* cell densities among brain regions for each condition. Tables 5, 6 show correlation coefficients and *P* values among brain regions and behaviors in relevant conditions.

**Table 1:** Pearson correlation coefficients of *cfos* staining in brain nuclei of *A. burtoni* males exposed to control conditions.

		Dl-g	nPPa	ATn	Dc-5	Dc-4	Vv	Vs	Vd
Dp	R	0.703	0.176	−0.042	0.116	−0.090	0.233	0.073	−0.522
	P	0.119	0.739	0.937	0.852	0.866	0.657	0.891	0.288
Dl-g	R		0.310	0.278	−0.620	0.218	0.404	0.338	−0.033
	P		0.550	0.594	0.265	0.678	0.428	0.512	0.950
nPPa	R			0.743	−0.269	−0.496	0.318	0.578	0.330
	P			0.901	0.662	0.317	0.539	0.230	0.523
ATn	R				−0.731	−0.553	0.237	0.060	0.053
	P				0.160	0.255	0.651	0.911	0.920
Dc-5	R					0.406	0.255	0.234	−0.052
	P					0.497	0.679	0.704	0.933
Dc-4	R						0.399	0.349	0.346
	P						0.433	0.498	0.502
Vv	R							0.522	0.080
	P							0.288	0.880
Vs	R								0.751
	P								0.086

*Data represent correlation coefficients (R) and *P*-values (P) from dominant males exposed to control conditions (*N* = 6). Data were used to create heat map in Figure 7A*.

**Table 2:** Pearson correlation coefficients of *cfos* staining in brain nuclei of *A. burtoni* males exposed to sexually-relevant unimodal visual signals.

		ATn	Vd	Vs	Vv	nPPa	Dc-5	Dl-g	Dp
Dc-4	R	0.243	0.360	−0.492	−0.037	−0.467	0.026	−0.543	−0.235
	P	0.694	0.552	0.400	0.953	0.428	0.968	0.345	0.703
ATn	R		0.419	−0.489	0.179	−0.101	−0.502	0.018	0.255
	P		0.408	0.325	0.734	0.849	0.310	0.973	0.626
Vd	R			0.504	0.282	−0.130	−0.582	−0.269	−0.455
	P			0.308	0.588	0.805	0.225	0.606	0.364
Vs	R				0.273	0.283	−0.223	−0.024	−0.573
	P				0.601	0.586	0.671	0.964	0.235
Vv	R					0.809	−0.375	−0.067	−0.523
	P					0.051	0.463	0.899	0.287
nPPa	R						−0.319	0.399	−0.146
	P						0.538	0.433	0.782
Dc-5	R							−0.552	−0.214
	P							0.256	0.684
Dl-g	R								0.755
	P								0.082

*Data represent correlation coefficients (R) and P-values (P) from dominant males exposed to visual only conditions (N = 6). Data were used to create heat map in Figure 7B*.

**Table 3:** Pearson correlation coefficients of *cfos* staining in brain nuclei of *A. burtoni* males exposed to sexually-relevant unimodal chemosensory signals.

		Dc-5	Dl-g	Vs	Vv	Vd	Dp	ATn	nPPa
Dc-4	R	0.635	0.435	0.609	0.107	0.016	−0.299	0.215	−0.655
	P	0.175	0.271	0.200	0.841	0.976	0.564	0.682	0.158
Dc-5	R		0.506	0.526	0.168	0.111	−0.144	−0.290	−0.546
	P		0.306	0.284	0.751	0.834	0.785	0.577	0.263
Dl-g	R			0.733	0.422	0.578	−0.212	−0.033	**−0.846**
	P			0.097	0.405	0.230	0.686	0.950	**0.034**
Vs	R				0.723	0.026	−0.663	0.351	−0.554
	P				0.104	0.961	0.151	0.495	0.254
Vv	R					−0.412	**−0.921**	0.075	−0.288
	P					0.417	**<0.01**	0.990	0.580
Vd	R						0.650	0.056	−0.375
	P						0.162	0.916	0.464
Dp	R							−0.081	0.246
	P							0.879	0.639
ATn	R								0.267
	P								0.609

*Data represent correlation coefficients (R) and *P*-values (P) from dominant males exposed to chemosensory only conditions (*N* = 6). Data were used to create heat map in Figure 7C. Bold indicates *P* < 0.05*.

**Table 4:** Pearson correlation coefficients of *cfos* staining in brain nuclei of *A. burtoni* males exposed to sexually-relevant multimodal visual-chemosensory signals.

		Dp	Dc-5	Dc-4	Vd	Dl-g	Vs	Vv	nPPa
ATn	R	−0.625	0.200	−0.169	−0.252	−0.078	0.131	0.019	0.396
	P	0.185	0.704	0.750	0.630	0.884	0.804	0.972	0.437
Dp	R		−0.173	−0.180	−0.081	−0.633	−0.648	−0.597	−0.752
	P		0.743	0.733	0.879	0.177	0.164	0.211	0.085
Dc-5	R			0.777	−0.466	−0.111	0.034	−0.057	−0.007
	P			0.69	0.352	0.835	0.949	0.915	0.989
Dc-4	R				−0.040	0.012	0.122	0.262	0.036
	P				0.940	0.981	0.819	0.616	0.946
Vd	R					0.221	0.582	0.786	0.580
	P					0.674	0.225	0.064	0.227
Dl-g	R						0.757	0.621	0.618
	P						0.081	0.188	0.191
Vs	R							**0.915**	**0.948**
	P							**0.010**	**0.004**
Vv	R								**0.892**
	P								**0.017**

*Data represent correlation coefficients (R) and P-values (P) from dominant males exposed to dual visual-chemosensory signals from females (N = 6). Data were used to create heat map in Figure 7D. Bold indicates P < 0.05*.

**Table 5:** Pearson correlation coefficients of *cfos* staining in brain nuclei and searching behavior of *A. burtoni* males exposed to control conditions and sexually-relevant uni- and multimodal visual-chemosensory signals.

		Control	Vision only	Chemosensory only	Vision and chemosensory
Dp	R	0.288	0.296	0.198	−0.324
	P	0.580	0.569	0.708	0.531
Dc-5	R	0.048	−0.165	0.304	−0.338
	P	0.939	0.755	0.558	0.513
Dc-4	R	−0.362	−0.638	0.389	−0.210
	P	0.481	0.247	0.445	0.690
ATn	R	−0.237	0.557	0.281	0.581
	P	0.651	0.251	0.589	0.227
Vd	R	**−0.918**	0.124	**0.850**	0.425
	P	**0.01**	0.815	**0.032**	0.401
Dl-g	R	−0.222	0.215	**0.852**	−0.297
	P	0.673	0.682	**0.031**	0.568
Vs	R	**−0.900**	−0.143	0.527	0.052
	P	**0.014**	0.786	0.282	0.922
Vv	R	−0.433	0.001	−0.005	0.265
	P	0.391	0.998	0.993	0.612
nPPa	R	−0.537	0.117	−0.637	0.340
	P	0.272	0.825	0.173	0.509

*Data represent correlation coefficients (R) and P-values (P) from dominant males exposed to each sensory condition (N = 6 for each condition). Data were used to create heat map in Figure 8A. Bold indicates P < 0.05*.

**Table 6:** Pearson correlation coefficients of *cfos* staining in brain nuclei and courtship behavior of *A. burtoni* males exposed to sexually-relevant unimodal visual signals and multimodal visual-chemosensory signals.

		Vision only	Chemosensory only
Dp	R	−0.225	−0.717
	P	0.668	0.109
Dc-5	R	−0.305	0.267
	P	0.556	0.609
Dc-4	R	−0.298	0.433
	P	0.627	0.391
ATn	R	0.009	0.114
	P	0.986	0.830
Vd	R	−0.090	0.493
	P	0.866	0.321
Dl-g	R	0.264	0.720
	P	0.613	0.107
Vs	R	0.180	**0.941**
	P	0.733	**0.005**
Vv	R	**0.896**	**0.918**
	P	**0.016**	**0.010**
nPPa	R	**0.976**	**0.882**
	P	**<0.001**	**0.020**

*Data represent correlation coefficients (R) and P-values (P) from dominant males exposed to visual only and dual visual-chemosensory signals (N = 6 for each condition). Data were used to create heat map in Figure 8B. Bold indicates P < 0.05*.

**Figure 9:**
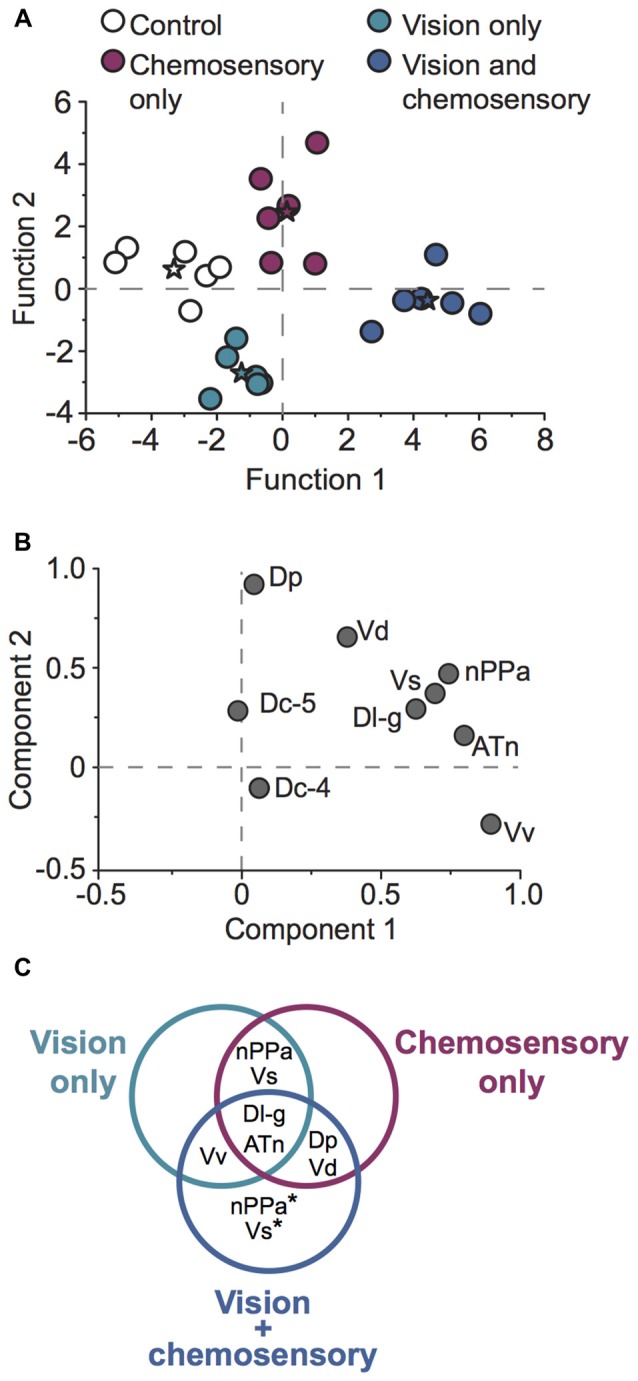
*A. burtoni* males exposed to unimodal and multimodal visual and chemosensory signals show distinct neural activation patterns. **(A)** Discriminant function analysis (DFA) correctly classified 100% of focal males into their respective sensory stimulus categories based on brain activation patterns alone. Stars represent group centroids of focal males exposed to control conditions (white), unimodal visual only (green), unimodal chemosensory only (purple), and multimodal visual and chemosensory signals (blue).** (B)** Principal component analysis (PCA) of *cfos* staining in socially-relevant and sensory processing brain regions. **(C)** Venn diagram summarizing neural activation in males exposed to unimodal visual only, unimodal chemosensory only, and multimodal visual and chemosensory signals from females. Brain regions that exhibited greater activation in at least one of the sensory conditions compared to control conditions are shown. Regions in overlapping circles indicate similar activation in each of the relevant overlapping conditions. Asterisks indicate regions that showed greater activation in the multimodal vision-chemosensory condition compared to the other unimodal sensory conditions. Regions that showed no change in activation compared to control conditions (Dc-4 and Dc-5) are not shown.

To examine whether patterns of neural activation could correctly classify individual focal males into their respective sensory stimulus condition, we performed canonical DFA and principal component analysis (PCA) on neural activation data for all investigated brain regions (Figure 9). DFA weights variable inputs (activation of brain regions) and determines if animals can be sorted into groups based on these variables, and identifies which variables may contribute to this sorting. Our DFA correctly classified 100% of animals into their respective groups (control, vision only, chemosensory only, vision and chemosensory; Figure 9A). Function 1 was driven positively by nPPa and Dp and explained 67.6% of the variance. Function 2 was driven positively by Vd and Dc-5 but negatively by nPPa and Vv, and accounted for 29.2% of the variance. Together functions 1 and 2 explained 96.8% of the variance and separated males into the four sensory stimulus groups based on neural activation patterns alone. PCA of *cfos* activation in examined brain regions produced two significant components driving variability in the data (Figure 9B; *N* = 24 animals; Kaiser-Meyer-Olkin measure of sampling adequacy = 0.577; Bartlett’s test of sphericity chi-squared = 101.693, *df* = 36, *P* < 0.001). Component 1 accounted for 42.8% of variance and was strongly weighted by ATn, Vv and nPPa. Component 2 accounted for 17.982% of variance and was loaded by Dp and Vd. Based on the regions driving each node, components 1 and 2 likely represent visual and chemosensory inputs, respectively. A summary of differences in neural activation in each region with each unimodal and multimodal sensory input (compared to control conditions) is shown in the Venn diagram of Figure 9C.

## Discussion

We investigated behavioral, physiological and neural responses of dominant *A. burtoni* males to uni- and multimodal visual and chemosensory signals from reproductively-receptive females. Our results show that males need sexually-relevant visual signals from females to engage in stereotypical courtship behaviors such as body quivers, tail waggles, and leads into the spawning territory. However, the number of courtship behaviors was greater when males were simultaneously exposed to visual and chemosensory signals from females, compared to either sensory signal alone. When a female visual signal was absent, males showed increased searching activity in response to female-conditioned water compared to control water, suggesting that these chemosensory signals may stimulate male motivation. Importantly, we also tested anosmic (olfactory ablated) males to demonstrate that this searching behavior is primarily mediated by the olfactory system rather than gustation. Using the immediate early gene *cfos* as a proxy for neural activation, we also revealed that decision and olfactory processing regions show differential activation when dominant males are exposed to visual and chemosensory signals together compared to exposure of either sensory signal alone.

### Behavioral and Physiological Responses to Uni- and Multimodal Signals

By examining behavioral responses to both uni- and multimodal visual-chemosensory signals, we show that male *A. burtoni* must see a female to perform courtship behaviors, but courtship is dramatically increased when chemosensory information is also available. It is well known that animals across taxa use multimodal signals for communication, particularly in courtship and reproductive contexts (Darwin and Prodger, 1998). A multimodal signal often benefits the receiver by allowing for better detection and localization of the signaler (Hasson, 1989), reduced habituation of individual signals (Todt and Fiebelkorn, 1980), and potential priming of one signal by another (Partan and Marler, 2005). The classification of multimodal signals into two broad categories, redundant and non-redundant, is based on the receiver’s response to each unimodal component separately and the response to the combined multimodal signal (Partan and Marler, 1999). In a simplified description, redundant signals are those that “mean the same thing” or result in the same response alone and together, while non-redundant signals “carry multiple messages” or result in altered responses (Moller and Pomiankowski, 1993; Johnstone, 1996; Partan and Marler, 2005). Based on these criteria, visual-chemosensory multimodal signals from gravid *A. burtoni* females are non-redundant because males’ behavioral responses to each unimodal signal and to the combined multimodal signal are all different, demonstrating that they convey different information. Vision alone elicits courtship behaviors while smell alone does not, however smell alone does induce increased searching behavior. In addition, dual visual-chemosensory signals result in increased courtship behavior compared to visual only conditions, while maintaining the searching behavior. This altered behavioral response from a multimodal signal is called “modulation,” and thus, visual-chemosensory signals in *A. burtoni* are classified as non-redundant modulatory signals. The processing of signals from multiple sensory modalities often results in responses that are larger than the sum of responses from the individual signals. This response profile, termed “super-additive response” is a generally common occurrence with even weak stimuli resulting in strong super-additive responses (Stein and Stanford, 2008; Angelaki et al., 2009). Thus, males likely get arousal and/or motivational information from chemosensory signals and visual signals elicit the courtship behaviors. In a similar study in *A. burtoni*, males exposed to the putative pheromone 17α-20β-dihydroprogesterone as a chemosensory signal showed no difference in the number of courtship displays when presented with and without a female visual signal (O’Connell et al., 2013). However, we used female-conditioned water that contains a “cocktail” of odorants as opposed to a single putative pheromonal compound. Indeed, specific behavioral and physiological responses to pheromones from conspecifics are typically due to a particular combination of compounds rather than a single odorant (Stacey, 2003; Derby and Sorensen, 2008). This mixed vs. single odorant application likely accounts for the different responses, especially since the identity of the pheromonal compounds released by *A. burtoni* are currently unknown.

We also examined male physiological responses to visual and chemosensory uni- and multimodal signals by measuring circulating 11-KT and E_2_ levels and found no difference across any condition. Similarly, males exposed to reproductive contexts in another study showed no difference in levels of 11-KT after exposure to visual and chemical stimuli (O’Connell et al., 2013). All males used in our study were highly dominant and territorial, meaning sustained high levels of these circulating hormones. The lack of context-specific stimulus-induced changes in our experiment may be due to circulating levels already being at or close to their physiological maximum (Parikh et al., 2006; Maruska and Fernald, 2010a; Maruska et al., 2013b; Maruska, 2015).

### Neural Activation in Response to Uni- and Multimodal Visual-Chemosensory Signals

Receiver behavior determines the use of true multimodal signaling in specific contexts (Partan and Marler, 2005), but there is a lack of information on how and where unimodal and multimodal signals are integrated in the brain to produce such behaviors (Partan, 2013). Using *in situ* hybridization for the IEG *cfos*, we examined neural activation patterns in social and olfactory-relevant brain regions as a result of uni- and multimodal signals. Some brain regions showed activation that was dependent on only one signal. For example, there was greater activation in Vv in males exposed to a visual signal from females, regardless of the presence of a chemosensory signal, while greater activation in Vd occurred in response to chemosensory signals regardless of vision.

Vv (homologous in part to the external globus pallidus (dorsal Vv) and mammalian septum (ventral Vv)) is well-known for its role in reproduction and courtship behaviors (Satou et al., 1984; Wullimann and Mueller, 2004; Ganz et al., 2012; Elliott et al., 2017). Component 1 of our PCA was driven strongly by Vv activation demonstrating the importance of sexually-relevant information to Vv. Neurons in Vv of the zebrafish also pool inputs from diverse mitral cells in the olfactory bulbs and respond more strongly to a mixture than to individual components of an odorant, suggesting that olfactory processing in this region may contribute to control of general behavioral or physiological state (Yaksi et al., 2009). One explanation for why there was not greater activation in Vv with chemosensory signals is that olfactory bulb projections to the medial olfactory terminal region (border of Vd and Vv; Sas et al., 1993), which contains GABAergic cells (Maruska et al., 2017) may inhibit activation of Vv cells, resulting in lower *cfos* cell density following exposure to chemosensory signals. Here, visual exposure to females resulted in greater activation in Vv, as well as an increased courtship response in males. While recent evidence supports a division of the Vv into dorsal and ventral regions with distinct homologs (see above), we did not distinguish them in this study. The route by which visual information may arrive at Vv in the cichlid is unknown, but tracing studies in zebrafish show inputs to the ventral telencephalon from visual centers such as preglomerular nuclei and the preoptic area (Rink and Wullimann, 2004). Here we provide additional evidence for the already well-documented role of Vv in decisions related to sexual behaviors.

The mammalian homolog of the teleost Vd is somewhat debated, but is considered in part to be the nucleus accumbens and/or striatal formation (O’Connell and Hofmann, 2011; Ganz et al., 2012; Elliott et al., 2017), both of which are involved in reward behavior. In teleosts, Vd is important for arousal (Forlano and Bass, 2011) and receives direct input from the olfactory bulbs (Meek and Nieuwenhuys, 1998). We previously showed that *A. burtoni* females have increased activation in Vd following reproductive interactions in full contact settings (when chemosensory signals were presumably released by males; Maruska and Fernald, 2012; Field and Maruska, 2017). Here, greater activation in Vd occurred when males were exposed to female-conditioned water regardless of whether a visual signal was present or not, and there was reduced activation in anosmic males. Further, activation in Vd was positively correlated with searching behavior (indicator of motivation) in chemosensory only conditions in intact males. Thus, our results further support Vd in integrating sexually-relevant chemosensory signals that stimulate arousal/motivation in males, similar to the nucleus accumbens in mammals (Becker et al., 2001; Portillo and Paredes, 2004; Hosokawa and Chiba, 2005).

Dp (homologous to the mammalian primary olfactory cortex) also showed increased activation when smell was present, and reduced activation in anosmic males, confirming its role in olfactory processing (Satou, 1990; Meek and Nieuwenhuys, 1998; Kermen et al., 2013). Further, component 2 of our PCA was most strongly driven by activation in Dp. More than just primary odor detection, Dp is implicated in odor memory and deciphering quality of complex odor mixtures (Yaksi et al., 2009; Mori, 2014). In zebrafish, Dp neurons establish representations of complex odor objects, potentially for use in the formation and recall of odor memories (Yaksi et al., 2009). In Mozambique tilapia (*Oreochromis mossambicus*) gene expression in Dp changes depending on the odorants, providing information on social context of the odorant (i.e., from dominant male, subordinate male, receptive female; Simões et al., 2015). Investigation of which genes may be up- or down-regulated in Dp of *A. burtoni* following stimulation with female-conditioned water would provide further information on how sexually-relevant olfactory information may be processed to produce specific social behaviors.

Two regions we investigated showed an additive response to multimodal visual-chemosensory signals: nPPa and Vs. This response suggests *integration* of sexually-relevant visual and chemosensory signals in these regions. nPPa is a sub-region of the pre-optic area (POA) which is widely viewed as a core brain center for reproduction and social behaviors across vertebrates (Forlano and Bass, 2011). It also plays a major role as a sensory integration center leading to motor and neuroendocrine responses in a variety of social contexts, including aggression, sexual arousal, and reproduction (Forlano and Bass, 2011). Thus, greater activation in nPPa with either of the sexually-relevant unimodal signals, and even greater activation with paired visual-chemosensory signals may be expected, but future studies that examine which neuronal phenotypes might be activated in different sensory conditions should provide further insights. Similarly, Vs (homologous to mammalian medial amygdala) is involved in processing the salience of sensory information (Gray, 1999; Newman, 1999), including sexually-relevant chemosensory signals (Kyle and Peter, 1982) making it essential for sexual motivation (reviewed in Forlano and Bass, 2011). Indeed, Vs is crucial for courtship and spawning in males of several fish species (Kyle et al., 1982; Satou, 1990), and receives visual, chemosensory, and acoustic information (Kyle et al., 1982; Gray, 1999; Butler and Maruska, 2016). Further, gene expression in developing zebrafish larvae identify Vs as homologous to the central amygdala (Ganz et al., 2012). In addition, Vs receives input from POA, Vd and Vv (Demski and Northcutt, 1983; Meek and Nieuwenhuys, 1998), each of which showed specific responses to uni- and multimodal visual-chemosensory signals in *A. burtoni*. Vs also has reciprocal connections with the olfactory bulbs (Demski and Northcutt, 1983; Forlano and Bass, 2011), and the greater activation observed in chemosensory only conditions was eliminated in anosmic males. Thus, the activation from multimodal visual-chemosensory signaling we observed further support Vs in processing sensory information that is important for courtship behavior in *A. burtoni* males.

In contrast to nPPa and Vs, ATn and Dl-g showed greater activation in response to at least one unimodal signal and the multimodal signal compared to control, and these responses were not different from one another. This suggests a broader role in mediating visual and chemosensory stimuli. The precise function of ATn (putative homolog in part of the mammalian ventromedial hypothalamus (VMH)) in teleosts is currently unknown, but this region has projections to POA, suggesting involvement in social behavior (Meek and Nieuwenhuys, 1998). In *A. burtoni* females, activation of ATn was increased following aggressive interactions (Field and Maruska, 2017), which is also observed in mammals (Kollack and Newman, 1992; Lin et al., 2011; Field and Maruska, 2017). In *A. burtoni* males, ATn is important in the transition between social statuses (Maruska et al., 2013a), as well as processing mechanosensory signals from lateral line stimulation during aggressive interactions (Butler and Maruska, 2016), thus implicating ATn in a variety of social behaviors. As well as mediating aggression, our data indicate a role in reproductive contexts. Similarly, sexually dimorphic neurons in the VMH of mice regulate both sexual and aggressive behaviors in males (Yang et al., 2013). In the current study, exposure to each reproductively-relevant sensory condition resulted in a similar activation response compared to control conditions, and component 1 of our PCA was driven strongly by ATn. However, ATn may not be explicitly involved in processing signals from specific sensory modalities, but rather mediate a more general response in courtship and reproduction. Examining neural activation with a different IEG, such as *egr-1*, may better identify regions involved in particular behavioral responses.

Dl-g showed greater activation in response to chemosensory only and multimodal signals in comparison to control conditions. Dl (putative homolog of the mammalian hippocampus) is involved in learning and memory (Rodríguez et al., 2002; Harvey-Girard et al., 2012; Elliott et al., 2017). In *A. burtoni* females, Dl-g had greater activation in response to social (reproductive and aggressive contexts) compared to non-social conditions (Field and Maruska, 2017). The general increase in *cfos* expression shown here may reflect a similar response to general social stimulation. Activation in Dl-g was positively correlated with searching behavior during exposure to unimodal chemosensory signals, but there is not currently any evidence to support Dl-g as an olfactory processing region. However, *A. burtoni* males must be able to locate females in order to spawn. In fact, males that successfully completed a spatial learning task that allowed them to access females showed greater activation in the Dl (Wood et al., 2011). Thus, correlation of activation in Dl-g with searching behavior during chemosensory only conditions may reflect some aspect of spatial cognition in males motivated to locate females and their territory shelters for spawning. Further, Dl has been implicated in pattern separation and completion in teleosts, and functions in a very similar way to the hippocampal circuits of mammals (Elliott et al., 2017). However, further studies investigating activation of other Dl sub-regions (in addition to Dl-g) are needed to better understand its involvement in social behavior and sensory processing, as well as examination of visual and chemosensory inputs to Dl areas.

In addition to examining neural responses in individual regions, we also performed Pearson correlations among brain regions in response to uni- and multimodal visual-chemosensory signals to gain a better understanding of the functional connectivity, if any, of these regions (Teles et al., 2015). In control and visual only conditions, no significant correlations were observed among any investigated brain nuclei. When only chemosensory signals were present, however, there were negative correlations between Dp and Vv, as well as between nPPa and Dl-g. In response to multimodal signals, nPPa, Vv and Vs were all positively correlated with each other. While true functional connectivity of these regions could not be completely elucidated (possibly due to low sample size of 6 for each condition), the different neural activity patterns demonstrate unique responses to sexually-relevant visual, chemosensory, and combined visual-chemosensory signals in male receivers. Further, these regions exhibit anatomical connectivity and have each been implicated in courtship and reproductive displays (Demski and Northcutt, 1983; Meek and Nieuwenhuys, 1998; Forlano and Bass, 2011).

While our primary focus was to examine which brain regions might be involved in reception and processing of visual-chemosensory information, we also performed correlations of *cfos* cell density in each region with searching and courtship behaviors in male receivers. In control conditions, Vs and Vd were both negatively correlated with searching behavior, while in chemosensory only conditions Dl-g and Vd were both positively correlated with searching behavior, suggesting that chemosensory inputs may be driving a motivational response in males. No correlations with searching behavior were observed for any brain region in the vision only and visual-chemosensory multimodal contexts. However, males perform courtship behaviors during both of these conditions, and the drive to engage in reproductive behaviors likely overpowers searching. In other words, if a male can see a female he will engage in courtship behavior rather than searching behavior. It should also be noted that *cfos* expression is more associated with the reception of sensory information, rather than specific behavioral outputs (Teles et al., 2015). Although visual only conditions resulted in no correlations between neural activation and searching behavior, courtship behavior positively correlated with activation in Vv and nPPa. While these regions both have well-established roles in courtship, our data suggests that Vv and nPPa are likely involved in processing *visual* signals, at a neural level above the primary visual input centers, to produce specific reproductive behaviors.

In multimodal conditions, activation in Vv, nPPa and Vs, which all positively correlated with each other, also positively correlated with courtship behavior. These regions are all parts of the proposed SDMN (O’Connell and Hofmann, 2011); Vv and Vs are shared between the social behavior network and mesolimbic reward system that makes up the SDMN, and nPPa is part of the social behavior network (Newman, 1999; O’Connell and Hofmann, 2011). As such, all of these regions have well-established involvement in reproductive and courtship behavior in fishes (Demski and Knigge, 1971; Macey et al., 1974; Kyle and Peter, 1982; Kyle et al., 1982; Satou et al., 1984; Satou, 1990). Further, we previously showed that *A. burtoni* females had greater activation in these same regions during reproductive contexts (Field and Maruska, 2017), demonstrating similar functions in reproduction across sexes. Here, the observed co-activation of these regions correlated with courtship behavior of males provides evidence of integration of visual and chemosensory information from gravid females that are vital to reproductive success.

## Summary

All animals must constantly integrate information from their environment with their own internal state to make important behavioral decisions. While we previously knew that visual and chemosensory signals were important for communication between sexes in *A. burtoni* (Maruska and Fernald, 2012; Field and Maruska, 2017), we show here that *combined* visual and chemosensory signals result in higher levels of male courtship behaviors, demonstrating a crucial role for chemosensory signals in reproduction. The behavioral responses in receiver males allow us to classify multimodal visual-chemosensory signals as non-redundant modulatory signals (Partan and Marler, 1999). By examining neural activation patterns with the IEG *cfos*, we identify decision centers involved in processing information from visual and chemosensory signals alone and together at an integration level above primary sensory processing. Vv and Vd, for example, both showed differential activation driven by one sensory modality, while ATn and Dl-g show greater activation in response to any and all sensory inputs. nPPa and Vs, however, show greatest activation with combined visual and chemosensory signals, suggesting sensory integration for behavioral decisions. By correlating neural activation in socially-relevant brain regions with courtship behaviors, we also show that activation in Vv, Vs and nPPa is associated with increased courtship behavior in males receiving multimodal visual-chemosensory signals from receptive females. Further, our DFA correctly classified 100% of males receiving no sexually-relevant signals (control conditions), visual signals only, chemosensory signals only, and multimodal visual-chemosensory signals based on neural activation alone. Thus, we demonstrate that multimodal visual-chemosensory signals are fundamentally different from either signal alone. These data provide insight on how different components of multimodal sensory inputs are received in the social brain, linked to essential behavioral outputs, and provide a framework for future studies on the evolution of sensory perception and multimodal signaling across species. This study, and others like it, will collectively help to better establish brain homologies and functional neural networks that shape context-dependent social behaviors.

## Author Contributions

KF, CM and KM had full access to the data and take full responsibility for the integrity of the data analysis, designed the experiments and wrote the manuscript. KF and CM performed the experiments and KF analyzed the data. KM provided funding, equipment and supplies. All authors reviewed and approved the final version of the manuscript.

## Conflict of Interest Statement

The authors declare that the research was conducted in the absence of any commercial or financial relationships that could be construed as a potential conflict of interest.

## Footnotes

^1^http://www.boris.unito.it/
